# Single-cell whole genome sequencing reveals no evidence for common aneuploidy in normal and Alzheimer’s disease neurons

**DOI:** 10.1186/s13059-016-0976-2

**Published:** 2016-05-31

**Authors:** Hilda van den Bos, Diana C. J. Spierings, Aaron S. Taudt, Bjorn Bakker, David Porubský, Ester Falconer, Carolina Novoa, Nancy Halsema, Hinke G. Kazemier, Karina Hoekstra-Wakker, Victor Guryev, Wilfred F. A. den Dunnen, Floris Foijer, Maria Colomé Tatché, Hendrikus W. G. M. Boddeke, Peter M. Lansdorp

**Affiliations:** European Research Institute for the Biology of Ageing (ERIBA), University of Groningen, University Medical Center Groningen, 9713 AV Groningen, The Netherlands; Institute for Computational Biology, Helmholtz Zentrum München, Ingolstädter Landstr. 1, 85764 Neuherberg, Germany; Terry Fox Laboratory, BC Cancer Agency, Vancouver, BC V5Z 1 L3 Canada; Section of Pathology, Department of Pathology and Medical Biology, University of Groningen, University Medical Center Groningen, 9713 AV Groningen, The Netherlands; Section Medical Physiology, Department of Neuroscience, University of Groningen, University Medical Center Groningen, 9713 AV Groningen, The Netherlands; Division of Hematology, Department of Medicine, University of British Columbia, Vancouver, BC V6T 1Z4 Canada

**Keywords:** Aneuploidy, Single-cell sequencing, Alzheimer’s disease, Brain, Neurons

## Abstract

**Background:**

Alzheimer’s disease (AD) is a neurodegenerative disease of the brain and the most common form of dementia in the elderly. Aneuploidy, a state in which cells have an abnormal number of chromosomes, has been proposed to play a role in neurodegeneration in AD patients. Several studies using fluorescence in situ hybridization have shown that the brains of AD patients contain an increased number of aneuploid cells. However, because the reported rate of aneuploidy in neurons ranges widely, a more sensitive method is needed to establish a possible role of aneuploidy in AD pathology.

**Results:**

In the current study, we used a novel single-cell whole genome sequencing (scWGS) approach to assess aneuploidy in isolated neurons from the frontal cortex of normal control individuals (n = 6) and patients with AD (n = 10). The sensitivity and specificity of our method was shown by the presence of three copies of chromosome 21 in all analyzed neuronal nuclei of a Down’s syndrome sample (n = 36). Very low levels of aneuploidy were found in the brains from control individuals (n = 589) and AD patients (n = 893). In contrast to other studies, we observe no selective gain of chromosomes 17 or 21 in neurons of AD patients.

**Conclusion:**

scWGS showed no evidence for common aneuploidy in normal and AD neurons. Therefore, our results do not support an important role for aneuploidy in neuronal cells in the pathogenesis of AD. This will need to be confirmed by future studies in larger cohorts.

**Electronic supplementary material:**

The online version of this article (doi:10.1186/s13059-016-0976-2) contains supplementary material, which is available to authorized users.

## Background

Aberrant chromosome copy numbers, aneuploidy, has been observed in the developing and adult human brain. However, the reported frequency of neuronal aneuploidy varies widely (up to 40 %, with an average of ~10 %) [1–3] with some studies reporting no aneuploid cells at all [4, 5]. Since neurons are post-mitotic, the number of methods to screen for aneuploidy is limited and most of the previous studies used interphase fluorescence in situ hybridization (FISH). Interestingly, several recent studies using single-cell whole genome sequencing (scWGS) consistently found low levels (2–5 %) of aneuploid neurons in the human brain [6–8]. Compared to interphase FISH, which is intrinsically noisy [9], scWGS has three important advantages: (1) all chromosomes in each single cell can be analyzed (in contrast to a maximum of four chromosome-specific probes for interphase FISH); (2) each chromosome is probed thousands of times per cell (thousands of unique reads per chromosome representing distinct chromosomal regions); and (3) the results are not affected by variable probe hybridization or artifacts related to tissue sectioning or other causes which can result in false-positive or false-negative results. These advantages make single-cell sequencing, at least in theory, a more robust method for detecting aneuploidy.

Interestingly, aneuploidy is thought to be involved in the pathogenesis of Alzheimer’s disease (AD), the most common form of dementia [10]. Several studies have reported an increased level of aneuploid cells in the brains of AD patients [1, 5, 11–15]. For example, some studies showed that extra copies of chromosomes 11, 17, 18, and 21 were more prevalent in neurons from AD patients compared to controls [5, 11–13, 15]. In contrast, other studies reported evidence for selective aneuploidy such as a tenfold increase in chromosome 21 aneuploidy [12] or a twofold increase in X chromosome aneuploidy [14]. That extra copies of chromosome 21 were repeatedly described in AD neurons is interesting in view of observations that individuals with Down’s syndrome (DS), who also have an extra copy of chromosome 21, are much more likely to develop AD and at an earlier age than euploid individuals [16]. Based on such observations, it was postulated that trisomy of chromosome 21 and the resulting extra copy of the amyloid precursor protein (*APP*) gene, located on chromosome 21, could contribute to the pathogenesis of AD. Indeed, mutations in *APP* are observed in patients with familial AD and are known to cause early onset AD [17]. In contrast, Thomas and Fenech, although finding high levels of aneuploidy in hippocampal cells for chromosome 17 and 21 (18 % and 12 % for chromosomes 17 and 21, respectively), found no difference in aneuploidy rates from brains of AD and controls [15], questioning the involvement of trisomy 21 and 17 in the pathogenesis of AD.

Since the reported rates of aneuploidy in AD brains are mostly based on interphase FISH studies and vary widely, we used scWGS to re-examine neuronal karyotypes in individuals with different stages of dementia to determine the frequency of aneuploidy in normal and AD brain. We developed a pre-amplification-free library preparation method and validated its ability to karyotype single cells by confirming the presence of three copies of chromosome 21 in single DS cells. We found very low levels of aneuploid neurons in control and AD brains. Also, no aneuploidy was found in non-neuronal cells of a control and AD sample. Collectively, these results show that aneuploidy is not common in normal and AD brain and thus unlikely to contribute to the pathogenesis of AD.

## Results and discussion

### Validation of the pre-amplification-free method of preparing libraries

**Fig. 1:**
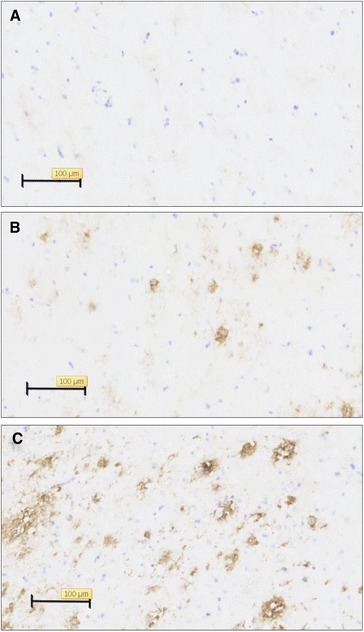
Examples of beta-amyloid plaque staining. Representative *images* of the area of the frontal cortex from where nuclei for sequencing were isolated of control individual (**a**) or AD patients with Braak stage III (**b**) or VI (**c**)

In this study, we used single-cell sequencing to assess the presence of aneuploid cells in the frontal cortex of normal postmortem brains and brains affected by AD (Braak stage III to VI). The presence of amyloid plaques in some of the brain samples classified with Braak stages III and VI was confirmed by amyloid β (Aβ) staining (Fig. 1). Nuclei were isolated from sections that were directly adjacent to sections with amyloid plaques. Single neuronal nuclei were sorted based on the nuclear neuronal marker NeuN as described previously [18]. scWGS libraries were prepared without whole genome pre-amplification (Additional file 1: Figure S1), reducing PCR amplification bias and thereby maintaining a more direct correlation between sequence reads and genome content. The distribution of reads across the chromosomes was used as a faithful indicator of the chromosome copy number. Since there is no pre-amplification step, a particular genomic location is expected to be represented in libraries only twice, one from each homolog of diploid individuals. Although the genomic coverage without pre-amplification is low, losses of genomic DNA during library preparation were typically found to be random. As a result, the distribution of reads mapping uniquely to the reference genome is rather even which allows accurate calls of chromosome copy number.

The copy number state of each chromosome was determined using an in-house developed algorithm called Aneufinder [19]. Briefly, this algorithm bins the mapped reads and uses a Hidden Markov Model (HMM) to predict the copy number state (i.e. monosomic, disomic, trisomic, etc.) for each bin. The most common state of a chromosome was assigned as the copy number for that chromosome. This means that when the majority of a chromosome is lost or gained, it is called monosomic or trisomic, respectively. Only libraries that passed the stringent quality metrics as determined by Aneufinder were used for further analysis: out of a total of 2664 single-cell libraries prepared for this study, 1632 libraries passed quality control (61 %). From these, we obtained on average 858,800 reads per library, of which 333,000 reads (with MAPQ >10) mapped to a unique location on the genome and library complexity was estimated to be 950,000 (see Additional file 2: Table S1 for more details). Importantly, the relatively shallow sequencing of the single-cell libraries is sufficient to determine chromosomal copy numbers. Higher coverage is possible by sequencing longer reads or fewer libraries per lane.

**Fig. 2:**
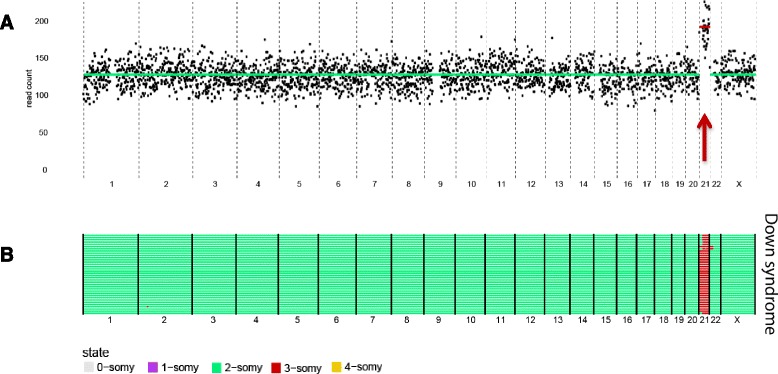
Trisomy of chromosome 21 is detected in DS cells. **a** Genome wide copy number plot of a single DS cell. *Arrow* denotes gain as identified by AneuFinder. **b** Genome wide copy number profile of a population of DS cells (n = 36). Each *row* represents a single cell with chromosomes plotted as columns. Cells are clustered based on the similarity of their copy number profile. Copy number states are depicted in *different colors* (see legend)

**Fig. 3:**
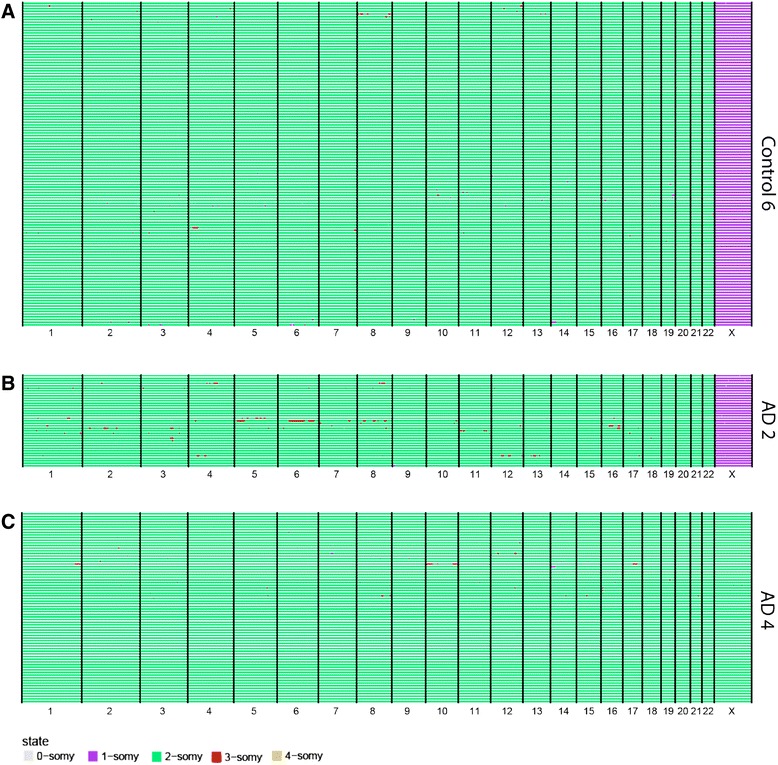
scWGS reveals no common aneuploidy in AD neurons. A representative genome wide copy number profile of a population of cells from control 6 (male, n = 120) (**a**) and two AD patients AD 2 (male, n = 37) and AD 4 (female, n = 72) (**b**) sample. Each *row* represents a single cell with chromosomes plotted as columns. Cells are clustered based on the similarity of their copy number profile. Copy number states are depicted in *different colors* (see legend)

To ensure that our approach faithfully and reproducibly annotates aneuploid events, we first validated our method by sequencing single neuronal nuclei isolated from a fresh frozen postmortem brain sample from an individual with DS. Indeed, in all 36 single-cell libraries, we detected three copies of chromosome 21, but no further aneuploidies (Fig. 2). In addition, two copies of chromosome X were called, as expected from this female individual. In contrast, all single neurons analyzed in our study isolated from male individuals had only one copy of the X-chromosome (Fig. 3a), further validating our single-cell-sequencing platform. Finally, scWGS data of several leukemic and solid tumor samples generated using this platform, revealed similar overall copy number variation (CNV) patterns as obtained by array comparative genomic hybridization (CGH) analysis ([19]; Paranita et al., personal communication), validating once more our approach to enumerate aneuploidy in single cells.

### Low level of aneuploidy in normal neurons

To assess the rate of aneuploidy in normal healthy brains we examined neurons from six control individuals without dementia. Of the 589 control neurons analyzed, all but four were euploid (Fig. 3a and Additional file 3: Figure S2A; Additional file 4: Table S2 and Additional file 5: Table S3). These four aneuploid cells were found in one control sample (n = 72): the first of which gained a copy of chromosome 18, the second cell lost a copy of chromosome 6, the third gained a copy of both chromosomes 4 and 16, and the fourth gained a copy of 13, 16, 21, and 22. Overall, the total prevalence of aneuploidy, cells with loss or gain of one or more chromosomes, in the control samples was 0.7 % (95 % confidence interval [CI]: 0.2–1.8 %, n = 589). The aneuploidy per chromosome, cells with loss or gain of a specific chromosome, was in the range of 0–0.34 % in Fig. 3a, Additional file 3: Figure S2, Additional file 4: Table S2, and Additional file 5: Table S3. The aneuploidy rates that we find in a normal brain are remarkably lower than reported by most other studies which used (interphase) FISH to detect aneuploidy [1–5]. For example, when comparing these results with the per chromosome aneuploidy rates reported by Iourov et al. [12] and Yurov et al. [14], we found significantly lower aneuploidy rates for all of the chromosomes analyzed in these studies (Mann–Whitney–Wilcoxon rank test, *p* <0.05 for chromosomes 1, 7, 11, 14, 17, 18, 21, and X in Iourov et al. [12] and for chromosomes 1, 7, 11, 16, 17, 18, and X in Yurov et al. [14]) (Additional file 5: Table S3). The FISH-based approach can yield noisy results, especially when used on tissue slides (as opposed to single cell suspensions) [9]. Our results are more in agreement with other recent studies that sequenced single neurons [6–8] and reported low rates (2–5 %) of aneuploid cells in normal brain. Similar to our analysis, these studies all analyzed human frontal cortical cells: McConnell et al. found one chromosome loss and two gains in 110 neurons (2.7 %) [6], Cai et al. reported four out of the 91 analyzed neurons to be aneuploid (4.4 %) [7], and Knouse et al. found two aneuploidies 89 cells (2.2 %) [8]. In summary, while our pre-amplification-free single-cell sequencing method faithfully detects aneuploidies such as trisomy 21 in a DS individual (Fig. 2) or X-chromosome monosomy in male cells (Fig. 3 and Additional file 3: Figure S2), it detects very low levels of aneuploidy in human adult neurons, indicating that previous FISH approaches may have overestimated aneuploidy levels in the human brain.

### Low level of neuronal aneuploidy in AD

While several groups have reported an increased level of aneuploidy in brains of AD patients compared to normal healthy brains, these observations were also based on FISH studies. Importantly, while our and other’s single-cell sequencing experiments [6–8] support that aneuploidy in a healthy brain has been overestimated in FISH studies, no single-cell sequencing data were available for AD patients’ neurons. Therefore, we examined 893 neurons from ten individuals with AD to investigate a potential role of neuronal aneuploidy in AD. In contrast to previous studies, we did not find evidence for increased aneuploidy in brains of AD patients (Fig. 3 and Additional file 6: Figure S3, Table 1, Additional file 4: Table S2, and Additional file 5: Table S3). In seven patients, no aneuploid cells were found, while in the other three patients, out of 261 cells a total of five aneuploid cells were found. Of the neurons from AD2 one cell had an extra copy of chromosome 6, of AD9 two cells lost either chromosome 3 or 21, and in AD10 one cell lost chromosome 12 and another gained chromosome 22. No evidence for increased rates of trisomy 21 in the assessed AD samples was found (Table 1 and Additional file 5: Table S3). The total neuronal aneuploidy rates in AD were comparably low as in control samples (0.6 %, 95 % CI: 0.2–1.3 %, n = 893). Again, these aneuploidy rates are significantly lower than reported previously (Mann–Whitney–Wilcoxon rank test, *p* <0.001 for chromosomes 1, 7, 11, 14, 17, 18, 21, and X in Iourov et al. [12] and for chr1, 7, 11, 16, 17, 18, and X in Yurov et al. [14]). Importantly, we can exclude detection issues, as we observed trisomy 21 in all neurons sampled from a DS control individual. Furthermore, we failed to detect selective gains of the other recurring AD chromosome gains reported in AD (e.g. trisomy 11 and 17). In fact, the few aneuploidies we did detect appeared to be random, as no particular chromosome loss or gain was found in more than two cells.

**Table 1:** Brain samples used and aneuploidy levels found per sample

Sample ID	Age	Sex	Braak stage	Libraries passing QC	Aneuploid cells
Control 1	69	M	0	81	0 (0 %)
Control 2	74	M	0	80	0 (0 %)
Control 3	79	F	I	108	0 (0 %)
Control 4	82	F	0	72	4 (5.56 %)
Control 5	84	F	I	128	0 (0 %)
Control 6	93	M	I	120	0 (0 %)
AD 1	64	F	VI	32	0 (0 %)
AD 2	66	M	IV	37	1 (2.70 %)
AD 3	73	F	V	63	0 (0 %)
AD 4	74	F	V	72	0 (0 %)
AD 5	76	F	III	118	0 (0 %)
AD 6	80	F	VI	125	0 (0 %)
AD 7	85	F	III	115	0 (0 %)
AD 8	85	F	VI	107	0 (0 %)
AD 9	91	F	III	109	2 (1.83 %)
AD 10	92	F	VI	116	2 (1.72 %)
Non-neuron control	84	F	I	63	0 (0 %)
Non-neuron AD	92	F	VI	51	0 (0 %)

Interestingly, a recent study using single-cell quantitative PCR reported the presence of local copy number gains, up to 12 copies, of the *APP* locus in AD neurons [20]. Even though the goal of our scWGS study was to examine whole chromosome copy number variation, we investigated this region more closely in AD neurons. No copy number gains of the *APP* locus were observed (Additional file 7: Figure S4).

**Fig. 4:**
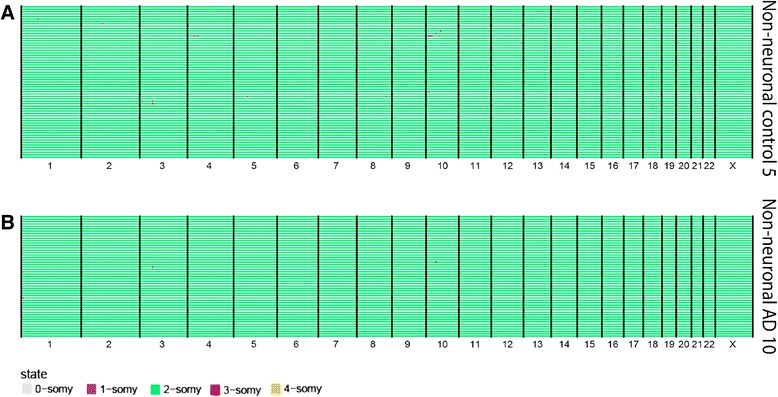
scWGS reveals no common aneuploidy in AD non-neuronal cells. Whole genome copy number profiles from non-neuronal cells from control 5 (female, n = 63) (**a**) and AD 10 (female, n = 51) (**b**). Each *row* represents a single cell with chromosomes plotted as columns. Cells are clustered based on the similarity of their copy number profile. Copy number states are depicted in *different colors* (see legend)

Although we do not observe a selective gain of chromosome 21 in neurons from AD patients, there is still a very compelling observation that individuals with DS develop early onset dementia with brain lesions similar as observed in AD patients [16]. As we focused our sequencing efforts on neurons only, we cannot rule out the possibility that aneuploidy in other cell lineages in the brain is involved in the pathogenesis of AD. Increasing evidence suggests an important contribution of the immune system to AD pathogenesis (reviewed in [21, 22]). Both microglia and astrocytes, the CNS-resident innate immune cells, have been shown to be involved in the onset and progression of AD. So far, no single-cell sequencing data are available of these types of cells from AD brains. Therefore, we also analyzed some non-neuronal (NeuN-negative) nuclei from a control (n = 63) and an AD (n = 51) sample by scWGS. We found no aneuploid cells in either of these non-neuronal controls (Fig. 4 and Additional file 5: Table S3). However, no clear distinction was made between the non-neuronal cells and further studies are needed to exclude a potential role of aneuploidy in cell types such as microglia or astrocytes in AD neurodegeneration.

Taken together, our analysis using scWGS reveals that the prevalence of aneuploid cells in the frontal cortex of control individuals and AD patients is very low.

## Conclusions

Many recent studies have reported a high prevalence of aneuploid neurons in AD brains, which led to the hypothesis that neuronal aneuploidy could be involved in the pathogenesis of AD. However, using a single-cell sequencing approach, we report low levels of aneuploidy both in neurons from AD patients as well as in neurons from non-diseased individuals. The level of neuronal aneuploidy in our study is much lower than was previously reported [1, 5, 11–15]. Nevertheless several lines of evidence strongly support our results. First, our method clearly detected trisomy of chromosome 21 in a DS sample and monosomy of chromosome X in all male samples showing the accuracy of our approach. Importantly, the validity of our scWGS method to study CNVs in leukemic and solid tumor samples was validated with array CGH in separate studies ([19], Paranita et al., personal communication). The study by Bakker et al. [19] also provides evidence that our technique can detect complex and partial aneuploidies. Second, the aneuploidy rates that we find in normal healthy neurons are more in line with recent findings from other single-cell sequencing studies [6–8]. Third, we analyzed over 1500 neuronal nuclei, which is to our knowledge the largest single cell sequencing dataset so far. Therefore, although more AD-affected brains should be assessed to exclude rare cases, our results do not support an important role for neuronal aneuploidy in the pathogenesis of AD.

## Materials and methods

### Tissue sources

Fresh-frozen postmortem brain samples from the frontal cortex were obtained from the Dutch Brain Bank and from the department of Pathology & Medical Biology of the University Medical Center Groningen (UMCG). In this study, samples from six non-demented controls (Braak stage 0–I) and ten AD patients (Braak stage III–VI) were used. Patient details are listed in Table 1. A fresh-frozen postmortem brain sample from an individual with DS served as a positive control for the detection of trisomy of chromosome 21.

### Amyloid plaque staining

Amyloid staining was performed to confirm the presence of amyloid plaques in the brain samples with Braak stage III and VI. Immunohistochemical staining with antibodies directed at Aβ (4G8, 1:500, Biolegend, 800702) was done on 10-μm frozen brain sections. The sections were pre-incubated in 0.3 % H_2_O_2_ for 30 min and blocked with 10 % normal horse serum in PBS with 0.3 % Triton-X100 (Sigma, 9002-93-1) for 30 min. Hereafter, sections were incubated overnight at 4 °C with the Aβ primary antibody in PBS containing 0.3 % Triton-X100 and 1 % normal goat serum. Unbound antibodies were washed away with PBS and sections were incubated for 1 h at room temperature with horse anti-mouse biotinylated secondary antibody (1:400, Vector, BA-2000). Finally, the sections were incubated in avidin-biotin-peroxidase complex (Vectastain ABC kit, Vector Laboratories, PK-6100) for 30 min and visualized with diaminobenzidine (Sigma, D-5637). Counterstaining was performed with cresyl violet for 2 min.

### Isolation of neuronal and non-neuronal nuclei

From each sample, ten sections of 50 μm or a small tissue block (~0.5–1 cm^2^), cut into pieces, was used for nuclei isolation. Neuronal nuclei isolation was performed as described previously [18] with minor modifications. Samples were kept on ice throughout the nuclei isolation procedure. In short, tissue sections were incubated in nuclear isolation buffer (10 mM Tris-HCl [pH 8], 320 mM sucrose, 5 mM CaCl_2_, 3 mM Mg(Ac)_2_, 0.1 mM EDTA, 1 mM dithiothreitol [DTT], and 0.1 % Triton X-100) for 5 min and filtered through a 70-μm filter using a plunger. Hereafter, nuclei were purified by ultracentrifugation (107,000 g for 2.5 h at 4 °C) through a dense sucrose buffer (10 mM Tris-HCl (pH 8), 1.8 M sucrose, 3 mM Mg(Ac)_2_, 0.1 mM EDTA, and 1 mM DTT). Supernatant was removed from the pelleted nuclei that were washed and resuspended in PBS containing 2 % bovine serum albumin (BSA) (PBS/2%BSA). Isolated nuclei were stored in nuclei storage buffer (50 mM Tris-HCl [pH 8], 5 mM Mg(Ac)_2_, 0.1 mM EDTA, 5 mM DTT, and 40 % glycerol) at –80 °C. On the day of sorting, nuclei were washed with PBS/2%BSA and resuspended in PBS/2%BSA containing an antibody directed against the nuclear neuronal marker NeuN (1:100.000, Millipore) and 4′,6-diamidino-2-phenylindole (DAPI; 10 μg/mL) and incubated for 45–60 min on ice. Single NeuN-positive or NeuN-negative and DAPI low nuclei were sorted into 5 μL freezing buffer (50 % PBS, 7.5 % DMSO, and 42.5 % 2X ProFreeze-CDM [Lonza]) in individual wells of a 96-well plate using MoFlo-Astrios (Beckman Coulter). Ninety-two single nuclei were sorted per plate. In two wells of each plate, ten nuclei were sorted as positive control and two wells without nuclei served as negative control. Plates were subsequently centrifuged at 500 g for 5 min at 4 °C before being gradually frozen to –80 °C in styrofoam boxes. Plates were stored at –80 °C until library preparation.

### Pre-amplification-free scWGS library preparation

Pre-amplification-free scWGS library preparation was performed using a modified version of a protocol described before [23]. All pipetting steps are performed using a Bravo Automated Liquid Handling Platform (Agilent Technologies, Santa Clara, CA, USA). All DNA purification steps between enzymatic reactions were performed using AMPure XP magnetic beads (Agencourt AMPure, Beckman Coulter, Brea, CA, USA). All enzymes used in the library preparation are obtained from New England Biolabs. After DNA fragmentation by micrococcal nuclease, end repair, and A-tailing of the DNA fragments was performed in one reaction mix including T4 DNA polymerase, T4 polynucleotide kinase, and Bst 2.0 warm start polymerase. End repair was performed at 25 °C for 30 min followed by the A-tailing reaction at 68 °C for 30 min. Subsequently without DNA purification, ligation reaction mixture containing T4 DNA ligase was added and Illumina PE forked adapters were ligated to either side of the DNA fragments. After clean up, the adapter containing DNA fragments were directly subjected to 17 cycles of PCR using Phusion High Fidelity DNA polymerase and custom barcoded primers. After PCR amplification, a final AMPure bead clean-up was performed and DNA was eluted in 6 μL elution buffer.

### Illumina sequencing

Since each single-cell library received a unique barcode, libraries can be pooled (multiplexed) and sequenced together. Per 96-well plate, the full volume (6 μL) of the single nuclei and negative controls were pooled together with 1 μL of the ten nuclei controls. Size selection was performed on a 2 % E-gel EX (Invitrogen) to isolate the mononucleosome fragments of approximately 280 bp (range of 200–400 bp). The DNA was eluted from the gel slices using Zymoclean gel DNA recovery kit (Zymo) according to the manufacturer’s protocol. The DNA quantity and quality were assessed using Qubit fluorometer (Invitrogen) and Bioanalyzer with High sensitivity chips (Agilent), respectively. For sequencing, clusters were generated on the cBot and single-end 50 nt reads were generated using the HiSeq2500 sequencing platform (Illumina, San Diego, CA, USA). In all runs, a pool of 192 libraries was sequenced on one lane of a flow cell.

### Data analysis

After demultiplexing, all reads were aligned to the human reference genome (GRCh37) using short read aligner Bowtie2 (version 2.2.4) [24] with default settings. The resulting BAM files were sorted using Samtools (version 0.1.18) [25] and duplicate reads were marked using BamUtil (version 1.0.3). Duplicate reads and ambiguous alignments (MAPQ >10) were filtered out using Aneufinder. Estimated complexity was calculated by downsampling the reads several times and determining the fraction of unique reads each time. Then the number of reads sequenced (seq_reads) was plotted against the number of unique reads (uni_reads) and a curve was fitted through the data points using the formula:

$$ \mathrm{u}\mathrm{n}\mathrm{i}\_\mathrm{reads} = \left({\mathrm{C}}_{\max }*\mathrm{s}\mathrm{e}\mathrm{q}\_\mathrm{reads}\right)/\left(\mathrm{K} + \mathrm{s}\mathrm{e}\mathrm{q}\_\mathrm{reads}\right), $$

where C_max_ was used as an estimation of the complexity of the library: the theoretical maximum unique reads in that library. K is the number of sequenced reads at which the number of unique reads is half of the library complexity. For subsequent CNV assessment, a custom pipeline was developed called AneuFinder [19]. Briefly, uniquely mapped reads are counted in, non-overlapping bins of variable size based on mappability with a mean of 1 Mb in size (for details: see Bakker et al. [19]). GC corrected uniquely mapped read counts, were used as observables in a Hidden Markov Model (HMM) with several possible hidden copy number states from nullisomy up to decasomy (ten copies). The emission distributions were modeled with a delta distribution for the nullsomy state and with negative binomial distributions for all other states, with means and variances that were fixed to multiples of the monosomy-state ones. Parameter estimates were obtained using the Baum–Welch algorithm. The final CNV calls were determined as the state with the highest posterior probability for each bin.

### Quality control

The quality of each library was assessed with several criteria: genomic coverage, bin-to-bin variation in read density (spikiness), entropy, number of ploidy state segments, and Bhattacharyya distance. Using the AneuFinder function “ClusterByQuality,” the libraries were clustered based on similarity of the quality control aspects (described in detail in Bakker et al. [19]). From each sample, the highest quality cluster, each of which had spikiness <0.21 and Bhattacharyya distance >1.0, were considered good quality libraries and used for aneuploidy calling.

### Statistics

Wilcoxon rank sum test was used to compare groups using wilcox.test in R. *P* values <0.05 were considered significant.

## Additional files

Additional file 1: Figure S1. Single-cell, pre-amplification-free library preparation protocol overview. *Schematic representation* of the library preparation protocol used: after sorting in 96-well plates, the DNA is fragmented using MNase. End repair and A-tailing are combined in one reaction and directly followed with ligation of the adapters. Barcodes are introduced during the PCR and after pooling and size selection the libraries are ready to be sequenced. (PDF 203 kb) Additional file 2: Table S1. Statistics of all single-cell libraries passing quality control. (XLSX 220 kb) Additional file 3: Figure S2. scWGS reveals no common aneuploidy in controls. Genome wide copy number profiles from single-cell libraries of five control individuals. Each *row* represents a single cell with chromosomes plotted as columns. Cells are clustered based on the similarity of their copy number profile. Copy number states are depicted in *different colors* (see legend). (PPTX 327 kb) Additional file 4: Table S2. Copy number states from all chromosomes of all libraries. (XLSX 141 kb) Additional file 5: Table S3. Aneuploidy rates per chromosome of all samples. (XLSX 13 kb) Additional file 6: Figure S3. scWGS reveals no common aneuploidy in AD. Genome wide copy number profiles from single-cell libraries of eight AD patients. Each row represents a single cell with chromosomes plotted as columns. Cells are clustered based on the similarity of their copy number profile. Copy number states are depicted in *different colors* (see legend). (PDF 1238 kb) Additional file 7: Figure S4. No local copy number variations at *APP* locus in AD neurons. UCSC genome browser view of the *APP* locus on chromosome 21 for all AD libraries (n = 893; *left*) and zoomed in for some libraries (*right*). (PDF 600 kb)

## Abbreviations

AD: Alzheimer’s disease
APP: amyloid precursor protein
Aβ: amyloid β
BSA: bovine serum albumin
CGH: comparative genomic hybridization
CI: confidence interval
CNV: copy number variation
DAPI: 4′,6-diamidino-2-phenylindole
DS: Down’s syndrome
FISH: fluorescence in situ hybridization
HMM: Hidden Markov Model
scWGS: single-cell whole genome sequencing
