# Erratum to: Single-cell whole genome sequencing reveals no evidence for common aneuploidy in normal and Alzheimer’s disease neurons

**DOI:** 10.1186/s13059-016-1015-z

**Published:** 2016-06-30

**Authors:** Hilda van den Bos, Diana C. J. Spierings, Aaron Taudt, Bjorn Bakker, David Porubský, Ester Falconer, Carolina Novoa, Nancy Halsema, Hinke G. Kazemier, Karina Hoekstra-Wakker, Victor Guryev, Wilfred F. A. den Dunnen, Floris Foijer, Maria Colomé-Tatché, Hendrikus W. G. M. Boddeke, Peter M. Landsdorp

**Affiliations:** European Research Institute for the Biology of Ageing (ERIBA), University of Groningen, University Medical Center Groningen, 9713 AV Groningen, The Netherlands; Institute for Computational Biology, Helmholtz Zentrum München, Ingolstädter Landstr. 1, 85764 Neuherberg, Germany; Terry Fox Laboratory, BC Cancer Agency, Vancouver, BC V5Z 1L3 Canada; Section of Pathology, Department of Pathology and Medical Biology, University of Groningen, University Medical Center Groningen, 9713 AV Groningen, The Netherlands; Section Medical Physiology, Department of Neuroscience, University of Groningen, University Medical Center Groningen, 9713 AV Groningen, The Netherlands; Division of Hematology, Department of Medicine, University of British Columbia, Vancouver, BC V6T 1Z4 Canada

## Erratum

After the publication of this work [1] it was noticed that there were errors in two author names.

The author names should appear as Aaron Taudt and Maria Colomé-Tatché.

This has been updated in the original article.
