# Supplementary figures and images for: Single-cell whole genome sequencing reveals no evidence for common aneuploidy in normal and Alzheimer’s disease neurons
Source: Genome Biol. 2016 May 31;17:116. doi: 10.1186/s13059-016-0976-2 (PMC4888403; doi:10.1186/s13059-016-0976-2)

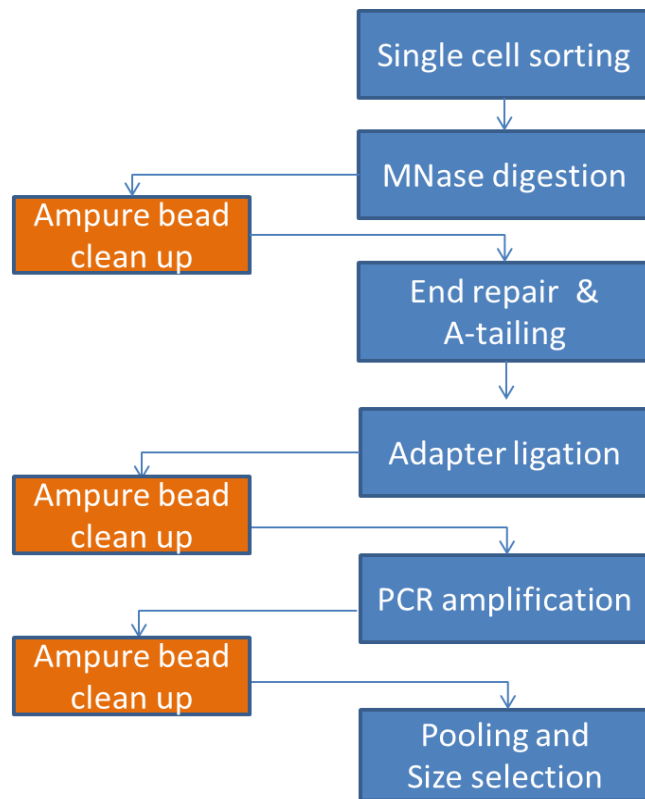

Supplement: Additional file 1: Figure S1. — Single-cell, pre-amplification-free library preparation protocol overview. Schematic representation of the library preparation protocol used: after sorting in 96-well plates, the DNA is fragmented using MNase. End repair and A-tailing are combined in one reaction and directly followed with ligation of the adapters. Barcodes are introduced during the PCR and after pooling and size selection the libraries are ready to be sequenced. (PDF 203 kb) [file 13059_2016_976_MOESM1_ESM.pdf]

## Slide 1
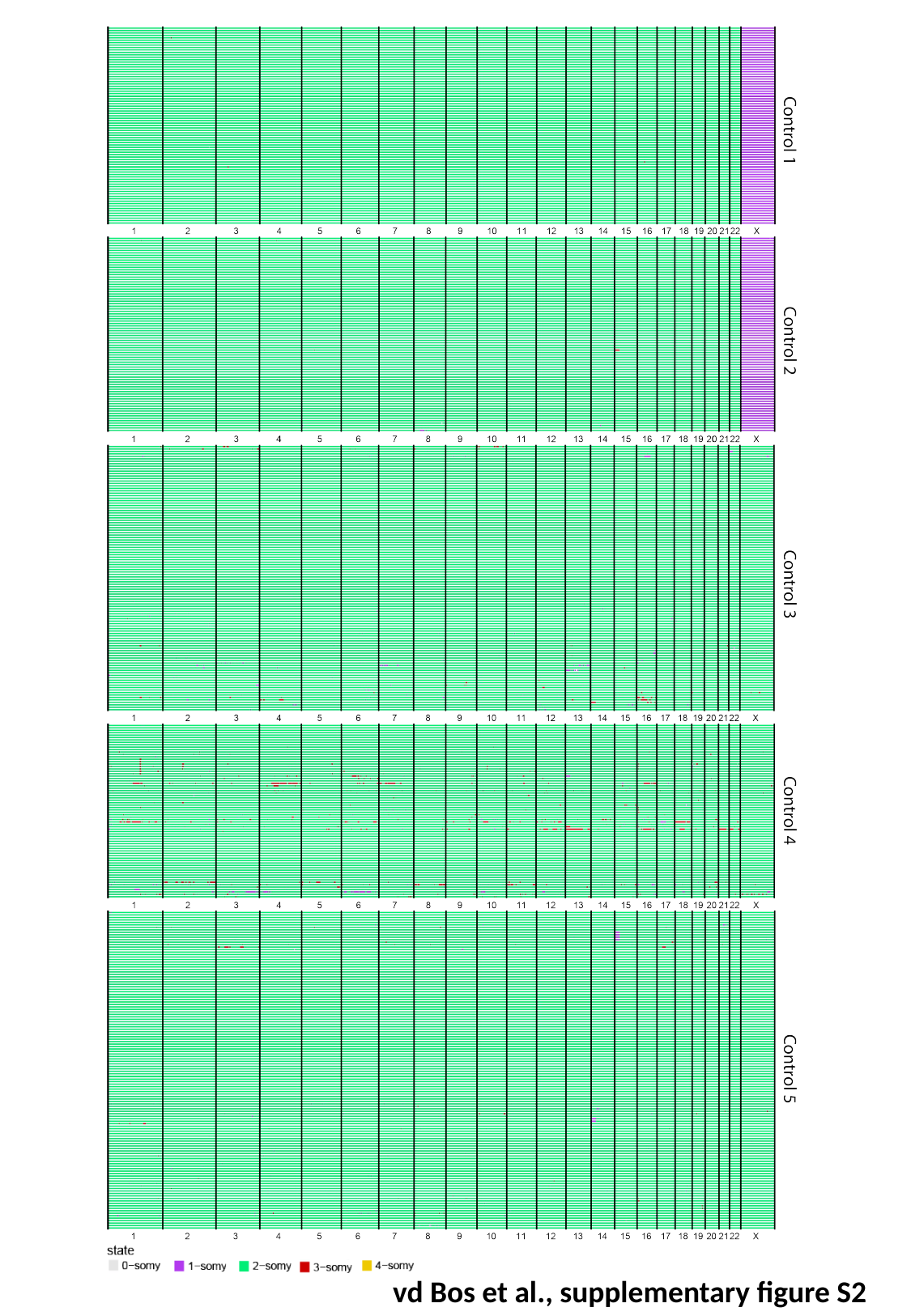

vd Bos et al., supplementary figure S2

Supplement: Additional file 3: Figure S2. — scWGS reveals no common aneuploidy in controls. Genome wide copy number profiles from single-cell libraries of five control individuals. Each row represents a single cell with chromosomes plotted as columns. Cells are clustered based on the similarity of their copy number profile. Copy number states are depicted in different colors (see legend). (PPTX 327 kb) [file 13059_2016_976_MOESM3_ESM.pptx]

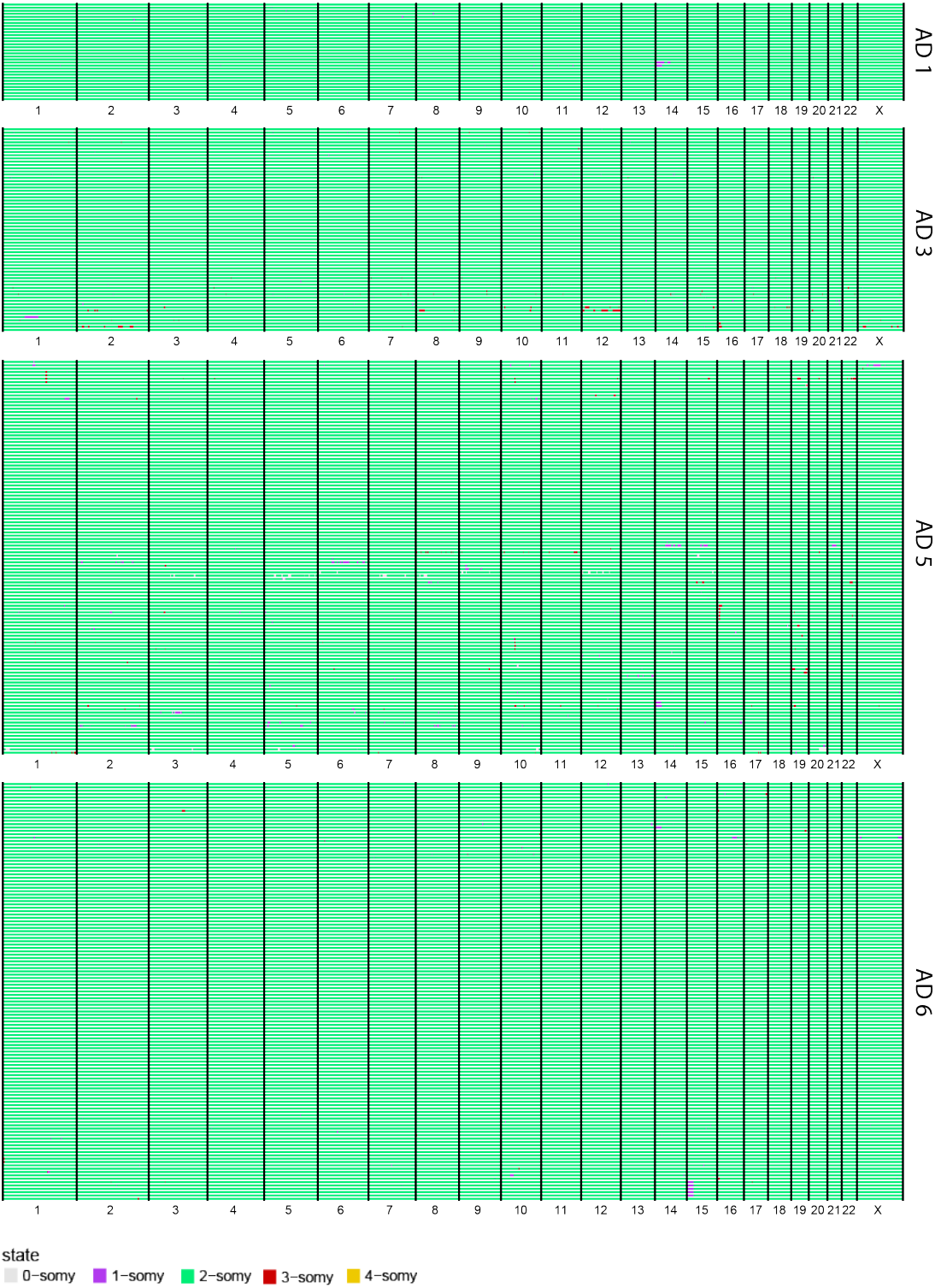

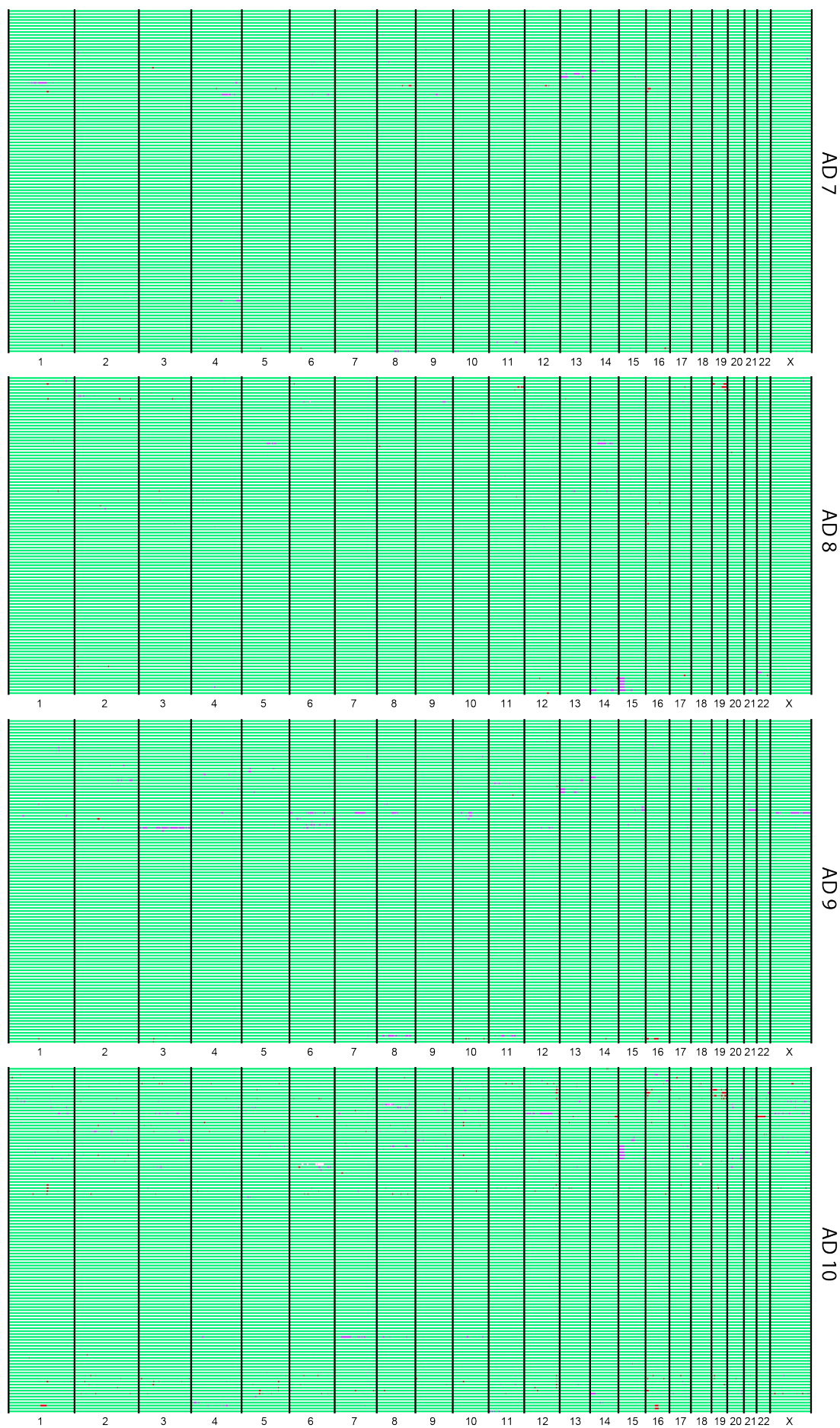

Supplement: Additional file 6: Figure S3. — scWGS reveals no common aneuploidy in AD. Genome wide copy number profiles from single-cell libraries of eight AD patients. Each row represents a single cell with chromosomes plotted as columns. Cells are clustered based on the similarity of their copy number profile. Copy number states are depicted in different colors (see legend). (PDF 1238 kb) [file 13059_2016_976_MOESM6_ESM.pdf]
