# Supplementary material for: Single-cell whole genome sequencing reveals no evidence for common aneuploidy in normal and Alzheimer’s disease neurons
Source: Genome Biol. 2016 May 31;17:116. doi: 10.1186/s13059-016-0976-2 (PMC4888403; doi:10.1186/s13059-016-0976-2)
Supplement: Additional file 7: Figure S4. — No local copy number variations at APP locus in AD neurons. UCSC genome browser view of the APP locus on chromosome 21 for all AD libraries (n = 893; left) and zoomed in for some libraries (right). (PDF 600 kb) [file 13059_2016_976_MOESM7_ESM.pdf]

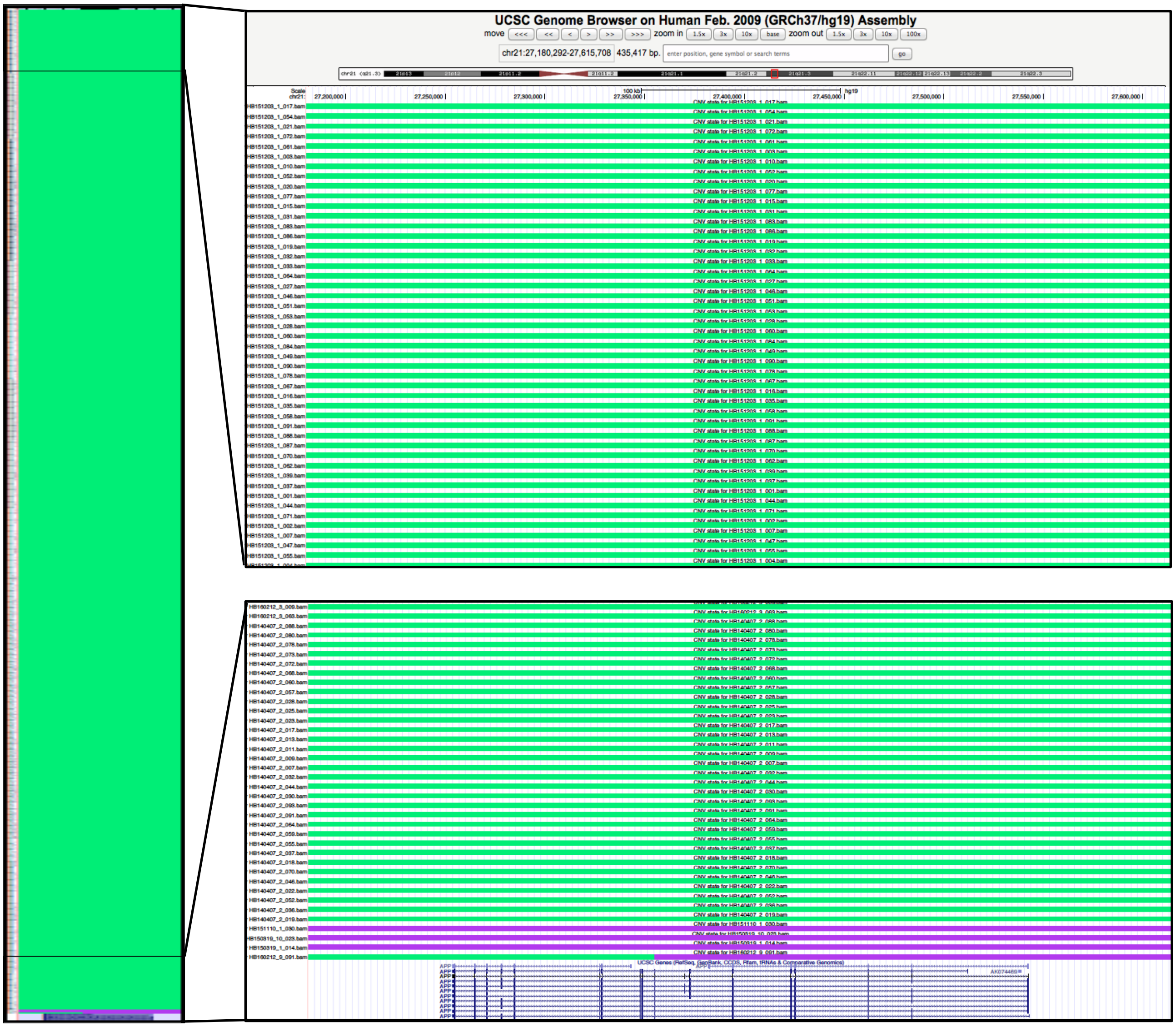

vd Bos et al., Supplementary figure S4
